# Deciphering breast cancer prognosis: a novel machine learning-driven model for vascular mimicry signature prediction

**DOI:** 10.3389/fimmu.2024.1414450

**Published:** 2024-08-06

**Authors:** Xue Li, Xukui Li, Bin Yang, Songyang Sun, Shu Wang, Fuxun Yu, Tao Wang

**Affiliations:** ^1^ Research Laboratory Center, Guizhou Provincial People’s Hospital, Guiyang, Guizhou, China; ^2^ NHC Key Laboratory of Pulmonary Immune-related Diseases, Guizhou Provincial People’s Hospital, Guizhou University, Guiyang, Guizhou, China; ^3^ Department of Breast Surgery, Guizhou Provincial People’s Hospital, Guiyang, Guizhou, China

**Keywords:** breast cancer, vascular mimicry, machine learning, immunotherapy, ispinesib

## Abstract

**Background:**

In the ongoing battle against breast cancer, a leading cause of cancer-related mortality among women globally, the urgent need for innovative prognostic markers and therapeutic targets is undeniable. This study pioneers an advanced methodology by integrating machine learning techniques to unveil a vascular mimicry signature, offering predictive insights into breast cancer outcomes. Vascular mimicry refers to the phenomenon where cancer cells mimic blood vessel formation absent of endothelial cells, a trait associated with heightened tumor aggression and diminished response to conventional treatments.

**Methods:**

The study’s comprehensive analysis spanned data from over 6,000 breast cancer patients across 12 distinct datasets, incorporating both proprietary clinical data and single-cell data from 7 patients, accounting for a total of 43,095 cells. By employing an integrative strategy that utilized 10 machine learning algorithms across 108 unique combinations, the research scrutinized 100 existing breast cancer signatures. Empirical validation was sought through immunohistochemistry assays, alongside explorations into potential immunotherapeutic and chemotherapeutic avenues.

**Results:**

The investigation successfully identified six genes related to vascular mimicry from multi-center cohorts, laying the groundwork for a novel predictive model. This model outstripped the prognostic accuracy of traditional clinical and molecular indicators in forecasting recurrence and mortality risks. High-risk individuals identified by our model faced worse outcomes. Further validation through IHC assays in 30 patients underscored the model’s extensive applicability. Notably, the model unveiled varying therapeutic responses; low-risk patients might achieve greater benefits from immunotherapy, whereas high-risk patients demonstrated a particular sensitivity to certain chemotherapies, such as ispinesib.

**Conclusions:**

This model marks a significant step forward in the precise evaluation of breast cancer prognosis and therapeutic responses across different patient groups. It heralds the possibility of refining patient outcomes through tailored treatment strategies, accentuating the potential of machine learning in revolutionizing cancer prognosis and management.

## Introduction

Breast cancer remains one of the leading causes of cancer-related mortality among women globally (1, 2), necessitating continuous advancements in diagnostic and prognostic technologies (3). Despite significant progress in understanding and treating breast cancer, the complexity of tumor biology, particularly the phenomenon of vascular mimicry (VM), presents ongoing challenges (4). VM, a process by which aggressive tumor cells mimic endothelial cells to form vasculogenic-like networks, has emerged as a critical factor in tumor growth, metastasis, and resistance to conventional therapies (5). This underscores the urgent need for innovative approaches to identify and target VM within the tumor microenvironment.

Recent advances in machine learning (ML) offer unprecedented opportunities to dissect complex biological processes, such as VM, through the analysis of large-scale datasets (6, 7). ML-driven models hold the promise of unveiling hidden patterns within tumor data, offering insights that could lead to the development of novel therapeutic strategies (8). However, the application of ML in understanding VM’s role in breast cancer and its potential as a prognostic marker remains underexplored.

This research aims to bridge this gap by developing and validating a novel ML-driven VM signature that can accurately predict breast cancer outcomes. Our study leverages multi-omics data to construct a detailed landscape of VM in breast cancer, highlighting its heterogeneity and pivotal role in tumor progression. By introducing a robust ML model capable of identifying VM-related signatures in breast cancer, our research contributes significantly to the field. In summary, our study not only addresses a critical gap in the current understanding of VM in breast cancer but also demonstrates the novel application of ML in uncovering potential therapeutic targets. As we move forward, it is imperative to explore these new frontiers in cancer research, leveraging cutting-edge technologies to combat one of the most challenging diseases of our time.

## Methods

### Data acquisition

Comprehensive gene expression profiles, mutation information, and essential clinical data were harvested from breast cancer cases in the TCGA database, with a focus on cases replete with survival data to ensure data integrity. Our methodology was further fortified by incorporating supplemental datasets retrieved from the GEO and MetaGxData databases (9). These datasets included GSE20685, GSE131769, GSE20711, GSE24450, GSE202203, GSE21653, GSE86166, GSE8532, GSE48391, and PNC, allowing for the substantiation of our VM-model across varied cohorts and enhancing the credibility of our research. The VM-related genes were retrieved from published study (10).

### Single-cell sequencing technique

Leveraging single-cell information from the GEO dataset GSE161529 (11), we began by filtering out genes not expressed in the dataset. Following normalization via Seurat’s “SC Transform,” we applied PCA and UMAP for dimensionality reduction and clusters were delineated using Seurat’s clustering functions. To ensure the quality of the dataset, we used DoubletFinder to identify and remove potential doublets (12), and stringent criteria were applied to exclude cells based on mitochondrial content or gene count. After rigorous quality checks, we retained a pool of 43,095 cells for detailed examination. The classification of cell types was achieved using Celltypist (13), which established a solid base for our subsequent analyses, identifying tumor cells via the copyKAT algorithm (14).

### Inter-cellular communication analysis

With the “CellChat” package in R (15), tailored CellChat objects were generated for each patient group. Employing “CellChatDB.human” as a reference, we proceeded with default parameters for analysis. The “mergeCellChat” function was instrumental in collating data to ascertain group-specific interaction dynamics.

### Functional analysis

Our analysis utilized the GO and KEGG databases to probe the variations in VM-related gene expression (16, 17). The Enrichplot package and clusterProfiler algorithm supported the Gene Set Enrichment Analysis (GSEA), focusing on differences between risk subgroups (18). A false discovery rate under 0.05 demarcated significant findings.

### Calculating the VM-score

Differential expression analysis on the TCGA-BRCA dataset helped us to discern gene activity distinctions in breast cancer compared to normal tissue. The ssGSEA and Ucell were employed on bulk and single-cell data respectively to derive a VM-score from these differentially expressed genes (DEGs) (19, 20). This score serves as a surrogate for VM activity in the cancer tissue. It’s essential to clarify that the VM-score does not directly quantify vascular structures but rather estimates activity from VM-associated gene expression. This analysis is pivotal as it juxtaposes the genetic activity profiles of cancerous and normal tissues, potentially reflecting the tumors’ vascular characteristics. Correlation through Spearman analysis with immune cell presence furnished a detailed insight into the role of the VM-score in the oncological context.

### Developing the VM prognostic model

Adopting the methodology introduced by Liu et al. (21), a VM prognostic model was created by applying ten diverse computational algorithms, each contributing uniquely to variable selection and dimension reduction, particularly RSF, LASSO, CoxBoost, and Stepwise Cox. The prognostic signature, derived from the TCGA-BRCA data, was evaluated across multiple datasets by its average C-index, revealing the model with the strongest predictive capacity for breast cancer outcomes. Our VM-model, evaluated through calibration curves, DCA, and multivariate Cox regression, stands as a testament to the study, acting as a robust instrument for outcome prediction in breast cancer. The risk scores were computed as follows:


$$riskcore=\sum_{i=1}^{n}\left(\beta_{1}\times Exp_{i}\right)$$

In this equation, ‘n’ represents the total number of VM genes included in the model, ‘Exp’ denotes the expression levels of the VM genes, and ‘β’ signifies the coefficients derived from the multivariate Cox regression model. The stratification of patients into different risk categories based on these scores allowed for a nuanced survival analysis. Utilizing external datasets validated the VM-model’s applicability across diverse patient populations. Kaplan-Meier analysis, with statistical significance set at a p-value below 0.05, was pivotal in establishing the model’s prognostic value across varied cohorts.

### Genomic alteration evaluation

Genetic variations between breast cancer patient groups stratified by risk level were explored harnessing the TCGA-BRCA repository to analyze mutation frequencies and copy number variations (CNA). Our study quantified tumor mutational burden (TMB) using data from TCGA’s raw mutation files, applying maftools to map mutations, particularly focusing on the most frequently altered genes (mutation rate > 5%). Using the deconstructSigs tool, we dissected patient-specific mutational patterns, uncovering four significant signatures (SBS1, SBS3, SBS11, SBS12) prevalent in the breast cancer cohort. Our scrutiny extended to chromosomal alterations, highlighting the five most affected regions by amplification or deletion. Particular attention was paid to four genes within the 3q26.32 and 19q13.32 regions. Through this detailed examination, we aim to elucidate the genetic factors that might influence the different risk levels and prognoses observed in breast cancer patient groups.

### Identifying TME disparities

In examining the varying immune cell infiltration in breast cancer patients stratified by VM-model, we leveraged the IOBR package to implement a battery of algorithms—MCPcounter, EPIC, xCell, CIBERSORT, quanTIseq, and TIMER—for a thorough and multi-perspective analysis (22–28). We further incorporated the ESTIMATE and TIDE indices to probe the immune microenvironment within the TME (29, 30). This investigation is essential for tailoring immunotherapeutic approaches and anticipating treatment outcomes for individuals with breast cancer. Additionally, we measured the presence of immune checkpoints to gain further understanding of the TME’s immune landscape. This metric is crucial for estimating the likelihood of patient responses to ICIs, integral to individualized cancer treatment regimens.

### Determining therapeutic targets and drugs

After eliminating duplicates, we curated an extensive list of 6,125 substances from the Drug Repurposing Hub (https://clue.io/repurposing). This endeavor aimed to forecast chemotherapy reactions and pinpoint potential therapeutic targets, hinging on Spearman correlations between risk scores and gene expression. We spotlighted genes linked to breast cancer prognosis, with emphasis on those presenting a correlation coefficient beyond 0.2 and P-value under 0.05. Our analysis included scrutinizing CERES scores against risk scores to isolate genes tied to poor outcomes using CCLE data (31).

To refine drug response forecasts, we harnessed the CTRP and PRISM resources, rich in drug and molecular data across cancer cell lineages. The pRRophetic package, employing a ridge regression model, was utilized to anticipate drug reactions using solid cancer cell line data, underpinned by 10-fold cross-validation (32).

We also embarked on a CMap analysis to discern optimal therapeutic agents for breast cancer, contrasting gene expression profiles across risk categories, and processing the top 300 genes through the CMap portal (https://clue.io/query). The inverse relationship between CMap scores and potential treatment efficacy in breast cancer emerged, guiding drug selection efforts.

### qRT-PCR and patient stratification

The 30 breast cancer tissues were obtained from patients who underwent surgery for *in situ* breast cancer at Guizhou Provincial People’s Hospital. Total RNA from breast cancer tissues was extracted with TRIzol (Invitrogen, USA). Subsequent cDNA synthesis and qRT-PCR were conducted using GoScript reverse transcriptase and Master Mix (Promega) as per the guidelines provided. The CFX96 Touch Real-Time PCR Detection System (BioRad, USA) was utilized for data acquisition. Gene expression normalization was done using GAPDH as the control, with the 2^-ΔΔCq^ method determining relative expression levels. Based on gene expression analysis, patients were classified into low or high-risk groups in line with the VM-model’s prescribed equation.

### Immunohistochemistry experiment

HE staining was performed on our collected breast cancer samples. Diagnostic assessment of the stained slides was independently carried out by two pathologists to maintain objectivity. Additional patient demographics and clinical attributes are detailed in **Supplementary Table S1**.

Immunohistochemistry (IHC) was performed on paraffin-embedded samples, applying techniques described in earlier studies (33, 34). The antibodies used are detailed in **Supplementary Table S2**. Protein expression was quantified using established scoring systems, with independent assessments made by two pathologists, mirroring protocols from previous research (34), to guarantee dependable evaluation.

## Results

### Deciphering the VM-related genes in breast cancer

**Figure 1:**
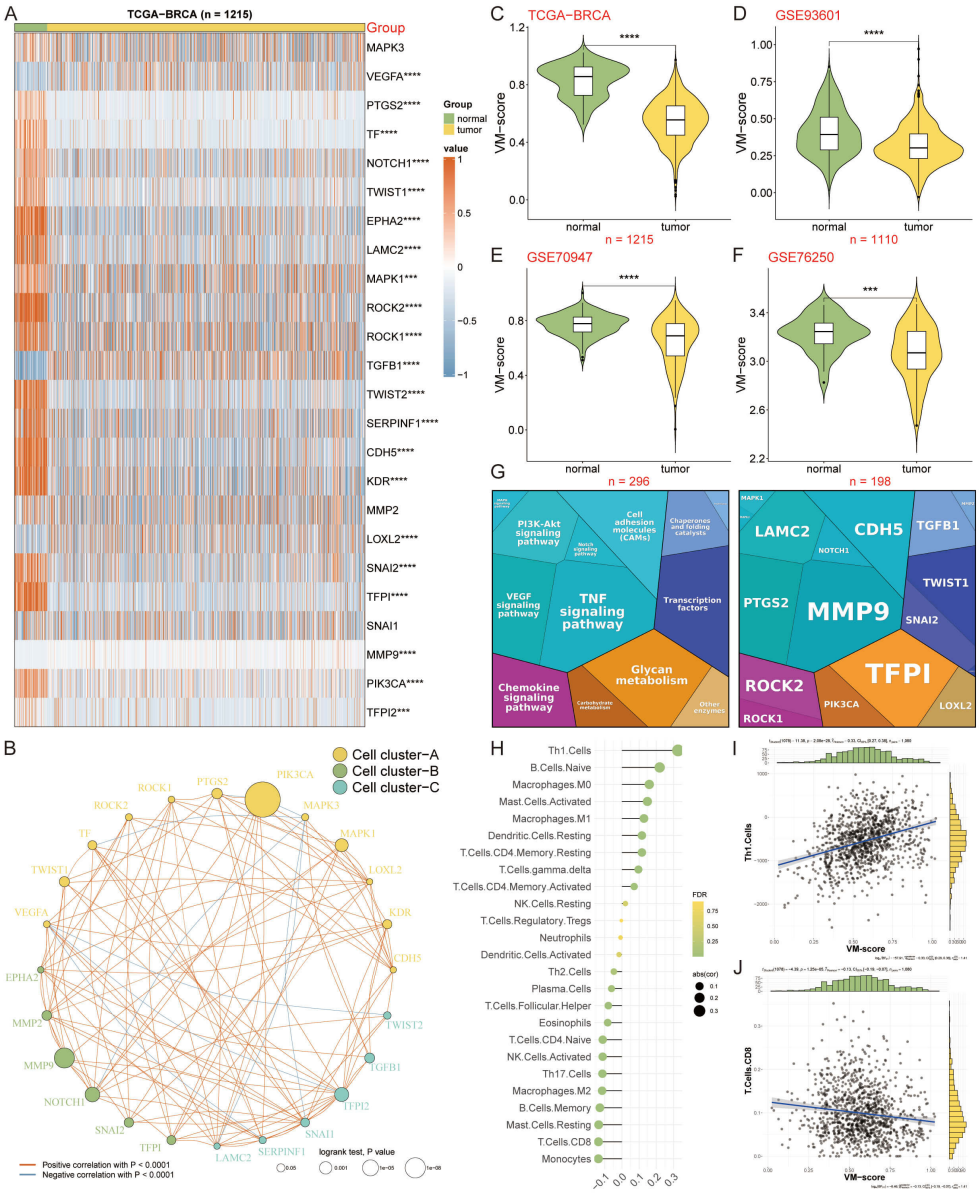
Deciphering the VM-related Genes in Breast Cancer. **(A)** Heatmap displaying differential expression of 24 key VM-related genes across normal individuals and breast cancer patients. **(B)** Regulatory network of VM-related genes organized into three clusters, illustrating interactions and correlations. **(C–F)** VM scores of breast cancer patients versus non-cancer individuals, calculated using the ssGSEA algorithm, with lower VM scores observed in patients across TCGA-BRCA and external datasets GSE93601, GSE70947, and GSE76250. ***P < 0.001. **(G)** Gene function map indicating the involvement of VM genes in critical pathways. **(H)** Correlation heatmap showing the relationship between VM scores and 25 types of infiltrating immune cells in the tumor microenvironment. **(I–J)** Scatter plots demonstrating significant correlations between VM scores and Th1 cell presence **(I)**, as well as CD8 T cell presence **(J)**, within the tumor microenvironment. ****P < 0.0001.

Our integrative heatmap analysis reveals distinct expression patterns of the first 24 genes associated with VM in breast cancer patients compared to normal individuals (**Figure 1A**). To elucidate the interplay between these VM regulators, we grouped them into three clusters and depicted their interactions in a regulatory network. Here, significant associations were uncovered; for example, NOTCH1 and TFPI from Cluster B exhibited a synergistic effect, in contrast to TF and MAPK3 from Cluster A, which demonstrated an antagonistic relationship. A particularly strong positive correlation was identified between PIK3CA and NOTCH1 (**Figure 1B**). To probe the connection between VM and breast cancer (breast cancer) progression, VM scores for each sample were computed utilizing the ssGSEA algorithm based on differentially expressed VM moderators. The compiled data revealed that breast cancer patients had significantly lower VM scores than those without breast cancer, corroborated by external datasets GSE93601, GSE70947, and GSE76250 (**Figures 1C–E**). Our gene function map highlights the involvement of VM-related genes in pathways like VEGF signaling, chemokine signaling, and cell adhesion (**Figure 1G**). Moreover, unique correlation pattern between VM-score and 25 infiltrating immune cells were observed (**Figure 1H**), as well as significant correlations with Th1 cells (**Figure 1I**) and CD8 T cells (**Figure 1J**) within the tumor microenvironment. These findings illustrate the complex regulatory landscape of VM in breast cancer, offering insights into potential biomarkers and therapeutic targets.

### Single-cell dissection of VM activity

**Figure 2:**
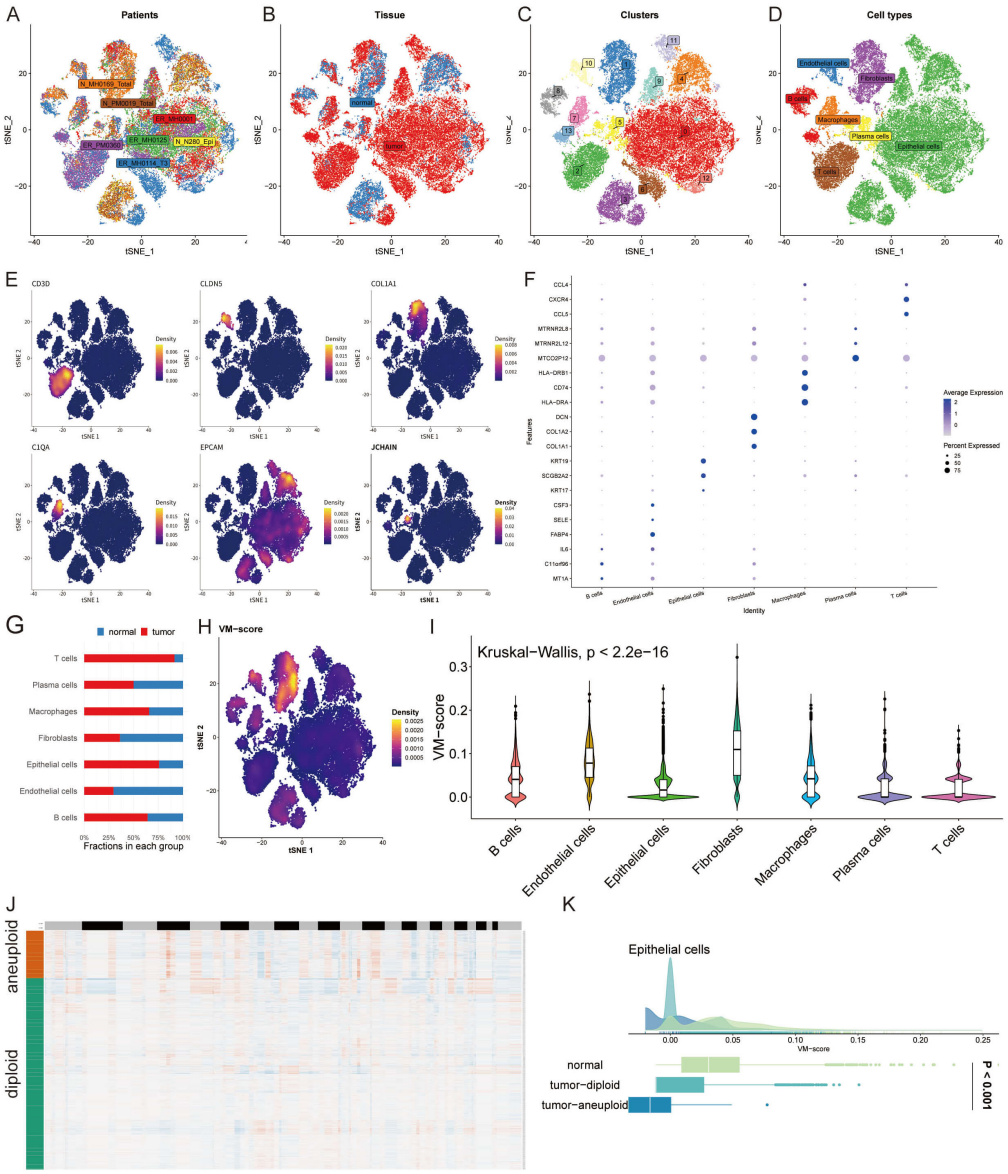
Single-Cell Dissection of VM Activity. **(A, B)** t-SNE plots illustrating patient samples and the distribution of normal and tumor tissues from an eight-patient cohort. **(C, D)** Identification of 13 unique cell clusters and annotation of seven distinct cell types within the tumor microenvironment. **(E, F)** Representative markers and differentially expressed genes for each identified cell type, highlighting the molecular diversity across clusters. **(G)** Bar graph comparing the proportion of T cells, B cells, macrophages, epithelial cells, plasma cells, fibroblasts, and endothelial cells in normal versus tumor tissue, evidencing a shift towards an immunosuppressive and pro-tumorigenic milieu in cancer. **(H)** Density plot of VM-score calculated using the UCell algorithm for individual cells. **(I)** Violin plots demonstrating the correlation of VM-score with seven annotated cell types, suggesting the differential involvement of these cells in VM processes. **(J)** Heatmap generated from copyKAT analysis indicating genomic variations. **(K)** Distribution of VM-score in epithelial cells comparing normal, tumor-diploid, and tumor-aneuploid groups, revealing the potential mechanistic links between aneuploidy and VM activity.

In a novel single-cell analysis, we enrolled eight patients and examined normal and tumor tissues (**Figures 2A, B**). This led to the identification of 13 distinct cell clusters, within which we annotated seven cell types (**Figures 2C, D**). Representative markers and top differentially expressed genes (DEGs) for each cell type were highlighted (**Figures 2E, F**). An analysis of the cell type proportions revealed a tumor-associated increase in T cells, B cells, macrophages, and epithelial cells, alongside a reduction in plasma cells, fibroblasts, and endothelial cells in tumor tissue (**Figure 2G**). Utilizing the UCell algorithm, we computed a VM-score for individual cells (**Figure 2H**) and estimated the correlation of VM-scores with the seven annotated cell types (**Figure 2I**). To specifically interrogate VM-score dynamics within cancer cells, we conducted a copyKAT analysis on epithelial cells, delineating genomic profiles (**Figure 2J**). Notably, VM scores were significantly elevated in aneuploid tumors compared to the normal group within epithelial cells, suggesting a potential link between aneuploidy and VM activity (**Figure 2K**).

### Elucidating cell-cell interaction dynamics in breast cancer

**Figure 3:**
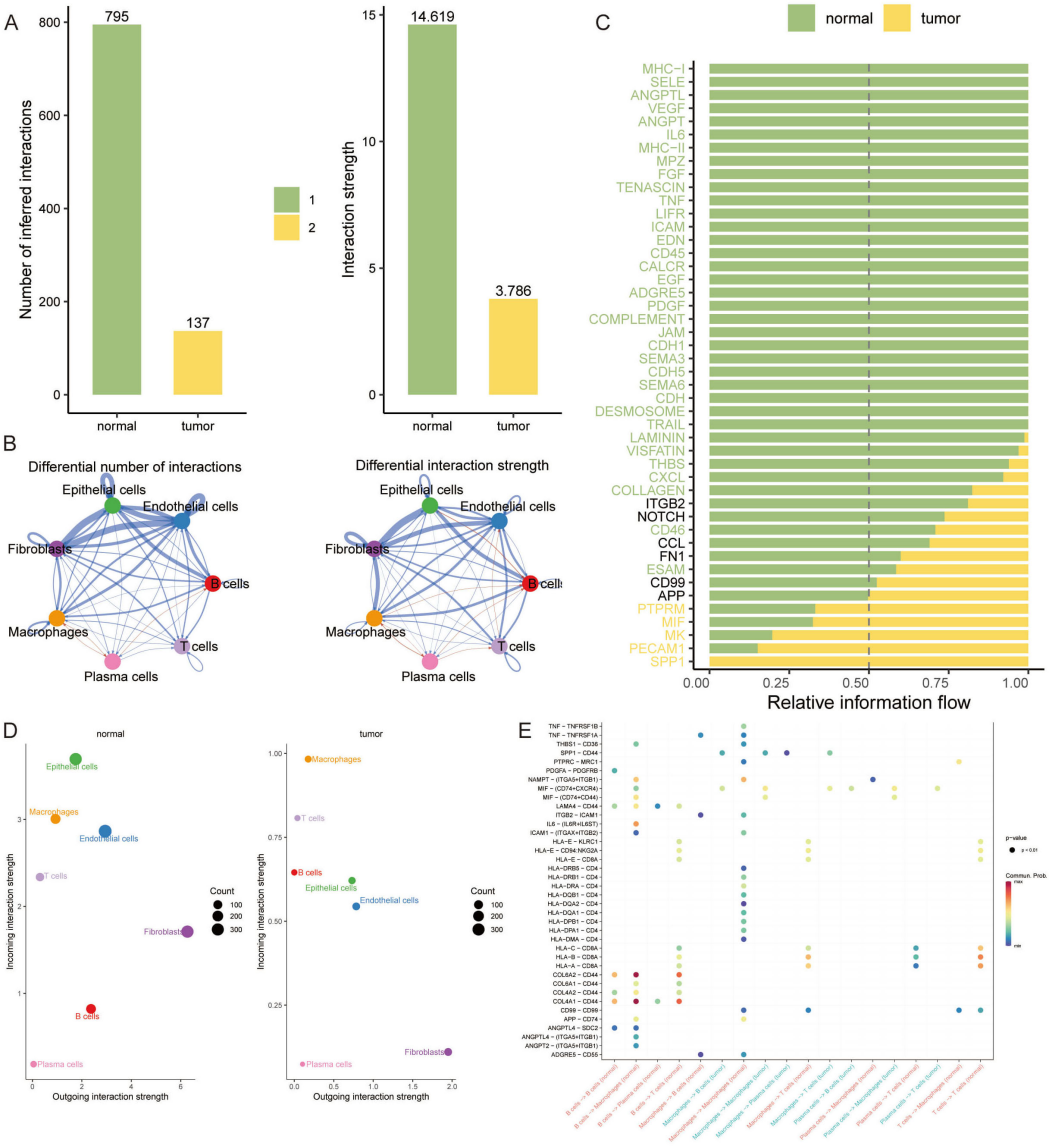
Elucidating Cell-Cell Interaction Dynamics in Breast Cancer. **(A, B)** Bar graphs illustrating the quantified cell-cell interaction frequencies among tumor and normal cell populations, revealing a reduced interaction in tumor cells, while plasma cells demonstrate increased connectivity, especially with B cells and macrophages. **(C)** Heatmap depicting the relative information flow through communication pathways. **(D)** Bubble plots representing the outgoing interaction strength of fibroblasts, which is consistent across conditions, and the prominently higher incoming interaction strength of epithelial cells in normal tissues. **(E)** Dot plot highlighting the significant tumor-specific interactions between COL1A1 and CD44, indicative of unique communication pathways within the tumor microenvironment.

A CellChat analysis was undertaken to decipher the intricate web of cell-cell interactions during breast cancer progression. Our analysis demonstrated a notable decline in the number and strength of interactions among tumor cells. In contrast, plasma cells showed an increase in interaction frequency and intensity, particularly with B cells and macrophages (**Figures 3A, B**). We further explored specific communication pathways and discovered that signaling via PTPRM, MIF, MK, PECAM1, and SPP1 was markedly more active in the tumor group compared to the normal group (**Figure 3C**).

Additionally, fibroblasts displayed a consistent outgoing interaction intensity in both normal and tumor tissues. Intriguingly, epithelial cells showed the highest incoming interaction intensity in normal tissue, highlighting their potential role in maintaining tissue homeostasis (**Figure 3D**). In the context of VM, our findings reveal a propensity for increased communication involving IIF and CD74+CXCR4 within each signaling pathway in the tumor group. Notably, the interaction between COL1A1 and CD44 emerged exclusively in the tumor group, underscoring a tumor-specific communication signature (**Figure 3E**).

### Constructing a VM prognostic model via machine learning

**Figure 4:**
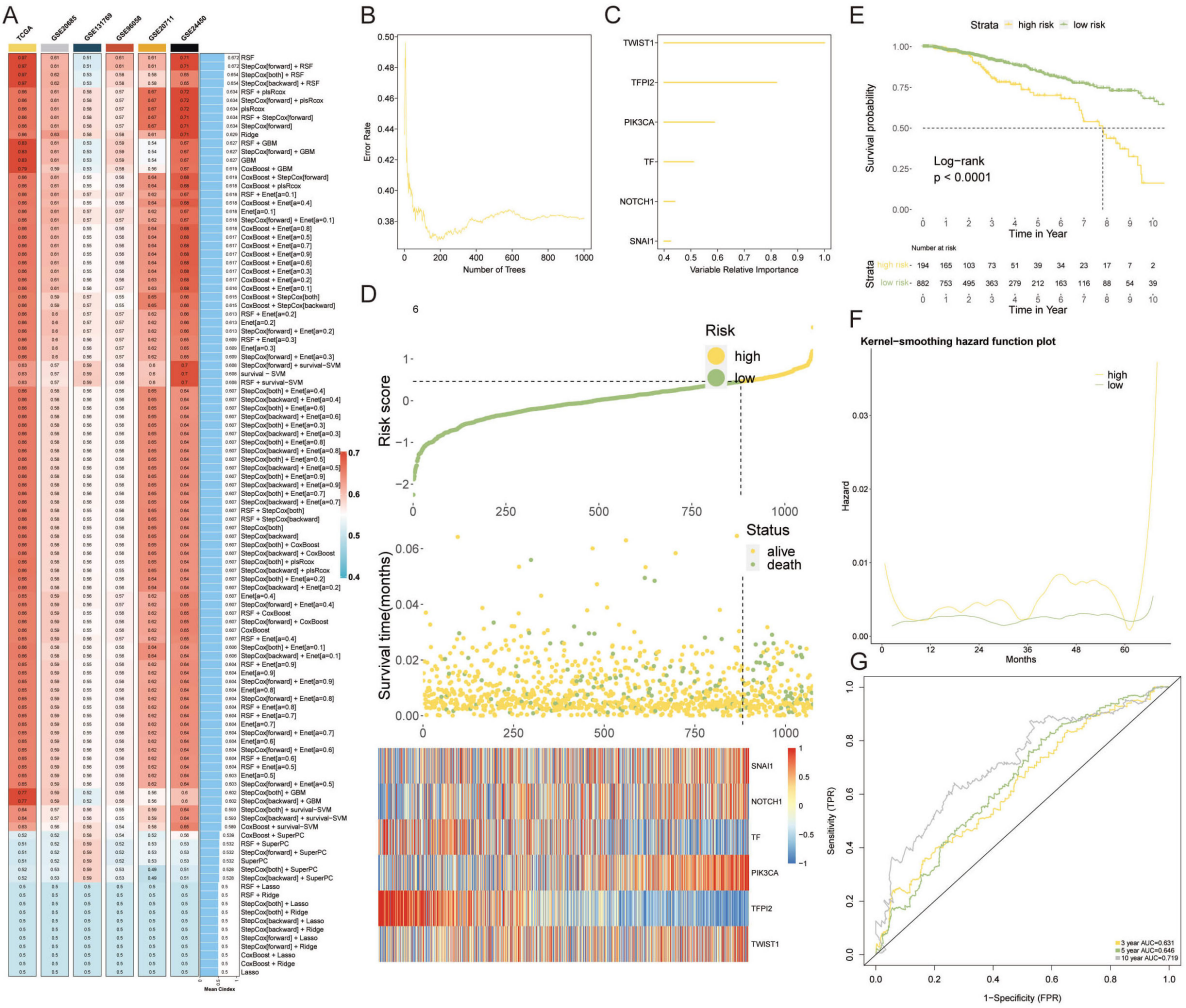
Constructing a VM Prognostic Model via Machine Learning. **(A)** The mean C-index values for 108 machine learning algorithms evaluated through ten-fold cross-validation across the TCGA-BRCA cohort and additional external cohorts, displaying the predictive accuracy of each model. **(B)** RSF variable importance plot demonstrating error rate in several different trees. **(C)** The importance of enrolled VM genes. **(D)** Heatmap detailing the expression patterns of the six prognostic genes across high-risk and low-risk patient groups, with notable overexpression in the high-risk category. **(E)** Kaplan-Meier survival curves contrasting the high-risk and low-risk groups, with a marked survival benefit observed for low-risk patients. **(F)** Kernel-smoothing hazard function plot indicating the differential recurrence risk over time between the high-risk and low-risk groups. **(G)** Time-dependent ROC curves assessing the predictive performance of the VM-model over 3, 5, and 10-year survival probabilities, with AUC values supporting the model’s prognostic utility.

To capitalize on VM-related genetic markers for prognostication in breast cancer, we engineered a VM-model by harnessing machine learning. A comprehensive suite of 108 algorithmic strategies was deployed, each rigorously vetted through ten-fold cross-validation within the TCGA-BRCA training cohort and five supplementary external cohorts. This approach enabled us to distill the algorithmic efficacy, quantified via the mean C-index across cohorts (**Figure 4A**). Subsequently, a Random Survival Forest (RSF) algorithm emerged as the cornerstone for our VM prognostic model. This model identified six key genes—TWIST1, TFPI, PIK3CA, TF, NOTCH1, and SNAI1—as significant prognosticators of breast cancer outcomes (**Figures 4B, C**). Risk stratification, predicated on the expression profiles of these genes, delineated patients into high-risk and low-risk categories. A heatmap depicted these genes’ expression levels, which were markedly elevated in the high-risk cohort (**Figure 4D**). Kaplan-Meier analysis underscored a pronounced survival advantage for the low-risk group (**Figure 4E**), while the kernel-smoothing hazard function plot intimated an escalated recurrence risk among the high-risk faction (**Figure 4F**). To corroborate these findings, we constructed a time-dependent receiver operating characteristic (ROC) curve, assessing the model’s predictive precision over 3, 5, and 10 years. The area under the curve (AUC) for these time points were calculated as 0.631, 0.646, and 0.719, respectively, validating the model’s prognostic strength (**Figure 4G**).

### Evaluating and validating the predictive efficacy of the VM-model

**Figure 5:**
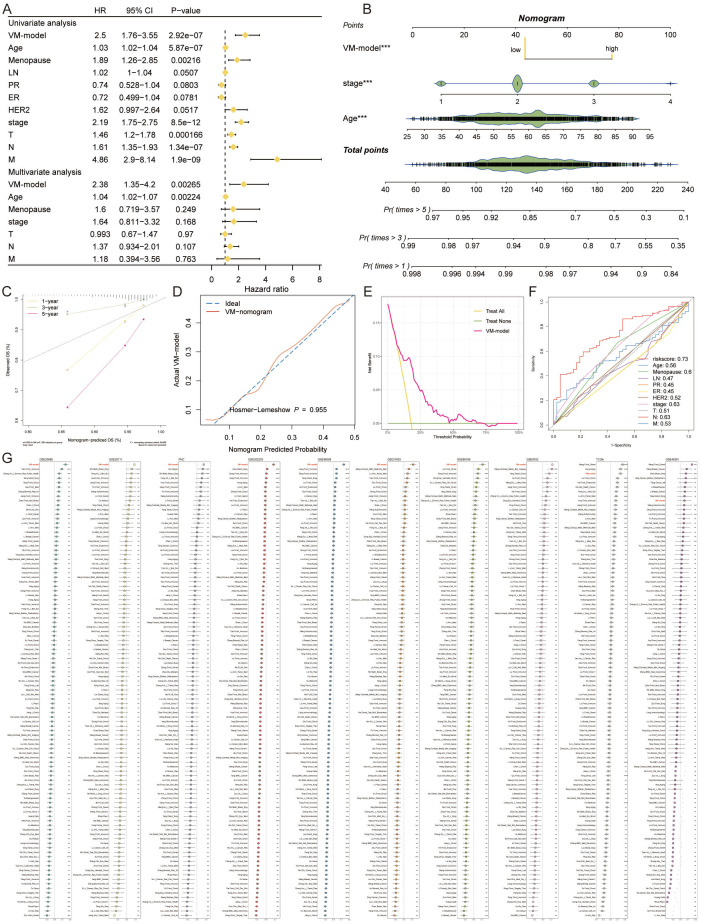
Evaluating and Validating the Predictive Efficacy of the VM-Model. **(A)** Forest plots from univariate and multivariate Cox regression analyses showing hazard ratios that establish the VM-model as a significant independent predictor of breast cancer prognosis. **(B)** A comprehensive nomogram integrating the VM-model with clinical factors to predict 1-year, 3-year, and 5-year survival probabilities, providing a personalized risk assessment tool. **(C)** Calibration curves for the nomogram demonstrating alignment with actual survival outcomes at 1, 3, and 5 years, validating the nomogram’s predictive accuracy. **(D)** DCA illustrating the clinical utility of the VM-model by comparing the net benefits of different treatment strategies. **(E)** Comparison of the VM-model’s predictive graph with ‘Treat All’ and ‘Treat None’ strategies, confirming the model’s utility in guiding clinical decisions. **(F)** ROC curves displaying the AUC for the VM-model’s risk score against other clinical factors, indicating superior prognostic performance. **(G)** A lengthy chart comparing the C-index of the VM-model with 100 other prognostic signatures across various cohorts, demonstrating the model’s top-ranking accuracy and robustness. *P < 0.05, **P < 0.01, ***P < 0.001, ****P < 0.0001.

The VM-prognostic model was rigorously assessed against established clinical factors using the TCGA dataset, with univariate and multivariate Cox regression analyses affirming its superior predictive performance (**Figure 5A**). This confirms the VM-model as a robust, independent prognostic factor for breast cancer. A nomogram incorporating the VM-model and clinicopathological factors was developed to forecast survival probabilities at 1-year, 3-year, and 5-year intervals (**Figure 5B**). Calibration curves for the nomogram’s 1-year, 3-year, and 5-year predictions exhibited high concordance with actual observed survival outcomes, underscoring the model’s accuracy (**Figure 5C**). The predictive values of the VM-model chart closely aligned with ideal observations, indicating no significant statistical discrepancy (**Figure 5D**). The model’s predictive graph surpassed the “Treat All” and “Treat None” benchmark curves, further confirming its reliable prognostic capabilities (**Figure 5E**).

In comparative analyses, the AUC value of the VM-model’s risk score eclipsed those of age, menopause status, lymph node involvement, progesterone receptor, estrogen receptor, HER2 expression, and TNM stages, in forecasting potential outcomes (**Figure 5F**). Extensive comparisons utilizing the C-index across the 10 cohorts further demonstrated the VM-model’s superior accuracy against 100 other signatures. Our VM-model consistently outperformed other models, ranking first in most cohorts, validating its exceptional robustness and prognostic relevance (**Figure 5G**).

### Multi-omics dissection of genetic variability through the lens of the VM-model

**Figure 6:**
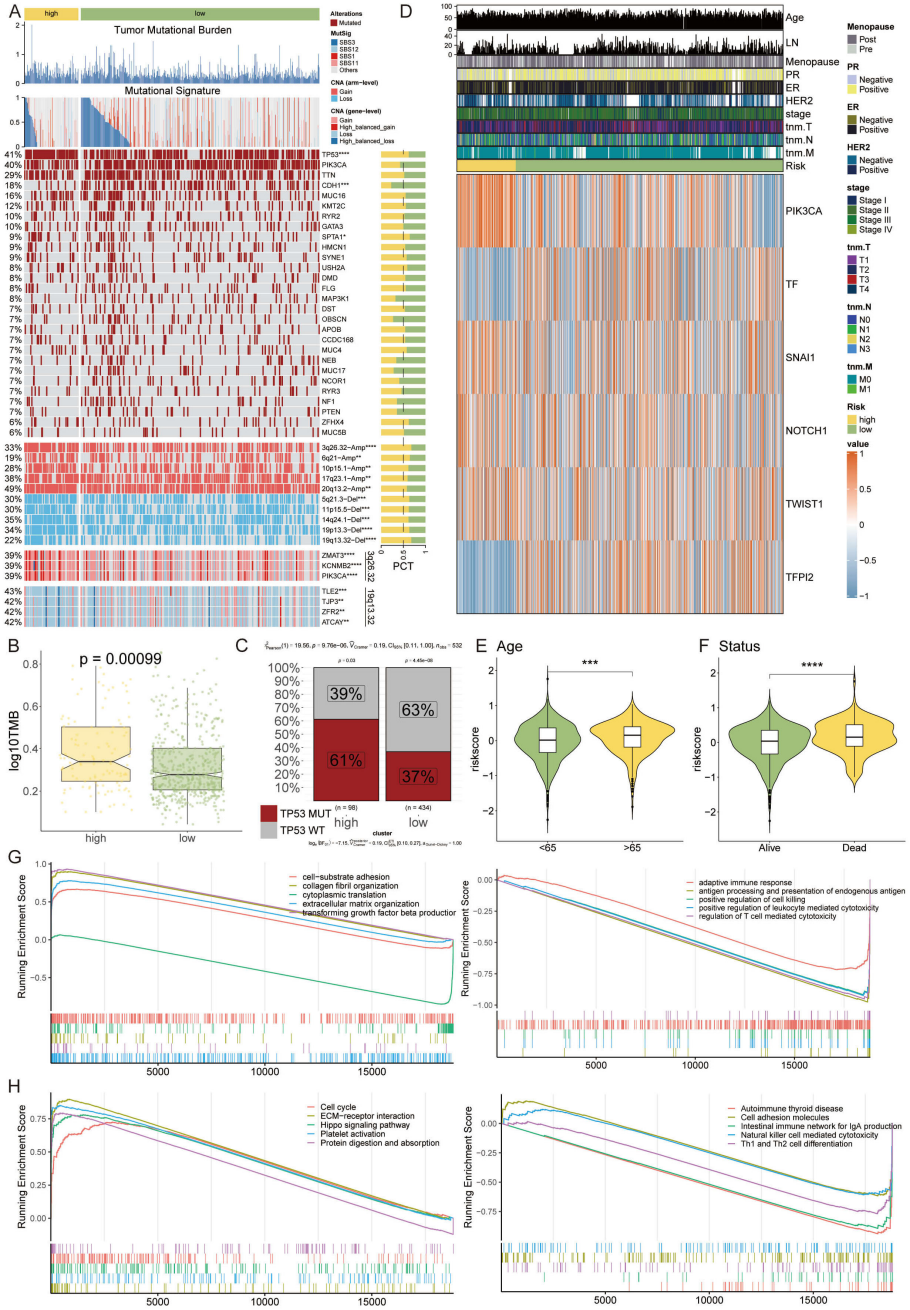
Multi-Omics Dissection of Genetic Variability through the Lens of the VM-Model. **(A)** A detailed heatmap showcasing the mutation profiles and copy number variations across the genome of high-risk and low-risk breast cancer patient groups. **(B)** Boxplots indicating TMB stratified by risk groups, with the high-risk group exhibiting a significantly elevated TMB. **(C)** Proportion charts showing the percentage of TP53 mutation occurrence within the high-risk and low-risk groups, emphasizing the genetic vulnerability of the high-risk group. **(D)** Heatmap representing the expression levels of six key VM modulator. **(E, F)** Violin plots correlating patient risk scores with age and survival status, suggesting an association between higher risk scores, advanced age, and poorer survival. ***P < 0.001. **(G, H)** GSEA plots illustrating the differential activation of various biological pathways between the risk groups, with the high-risk group showing suppression of immune response-related genes and an upregulation of genes involved in the cell cycle and extracellular matrix organization. *P < 0.05, **P < 0.01, ****P < 0.0001.

We embarked on a multi-omics interrogation of genomic diversity within the context of the VM-prognostic model, scrutinizing mutation profiles and copy number variances (**Figure 6A**). Our study highlighted the significant difference in TMB between the two VM groups, with the high-risk group exhibiting a markedly elevated TMB (p = 0.00099). This increased TMB is indicative of a higher genetic instability in high-risk tumors, which may contribute to their aggressive behavior and poor prognosis (**Figure 6B**). Our findings further elucidated that tumor suppressor genes TP53 and PIK3CA underwent mutations with a greater rate in the high-risk group than in the low-risk cohort. Additionally, our comparative analysis of copy number alterations (CNAs) underscored a significant amplification or deletion in the high-risk group, notably at chromosomal hotspots such as 3q26.32, 20q13.2, and 10p15.1, coupled with deletions at 5q21.3, 11p15.5, 19p13.3, and 19q13.32. These variations were substantiated by the conspicuous amplification of oncogenes ZMAT3, KCNMB2, and PIK3CA at 3q26.32, and the pronounced deletion of tumor suppressor genes TLE2, TJP3, ZFR2, and ATCAY at 19q13.32. The high-risk group was then characterized by a significantly augmented tumor mutational burden (TMB), with TP53 mutations presenting a marked increase compared to the low-risk group, reinforcing its genetic susceptibility (**Figure 6C**).

The heatmap analysis unveiled differential expression patterns of six pivotal VM modulators, among which TF and TFPI2 were distinctly upregulated in the high-risk category (**Figure 6D**). Assessing risk in relation to patient age and survival status disclosed a trend where higher risk scores were associated with advanced age and decreased survival (**Figures 6E, F**). Through functional annotation and gene enrichment assessments, we discerned an inhibited immune response concurrent with an activation of cell cycle and extracellular matrix organization pathways in the high-risk group (**Figures 6G, H**). This comprehensive analysis provides a nuanced understanding of the molecular underpinnings that could be instrumental in the progression of breast cancer, laying a foundation for potential therapeutic interventions.

### Delineation of the immune infiltration landscape in breast cancer

**Figure 7:**
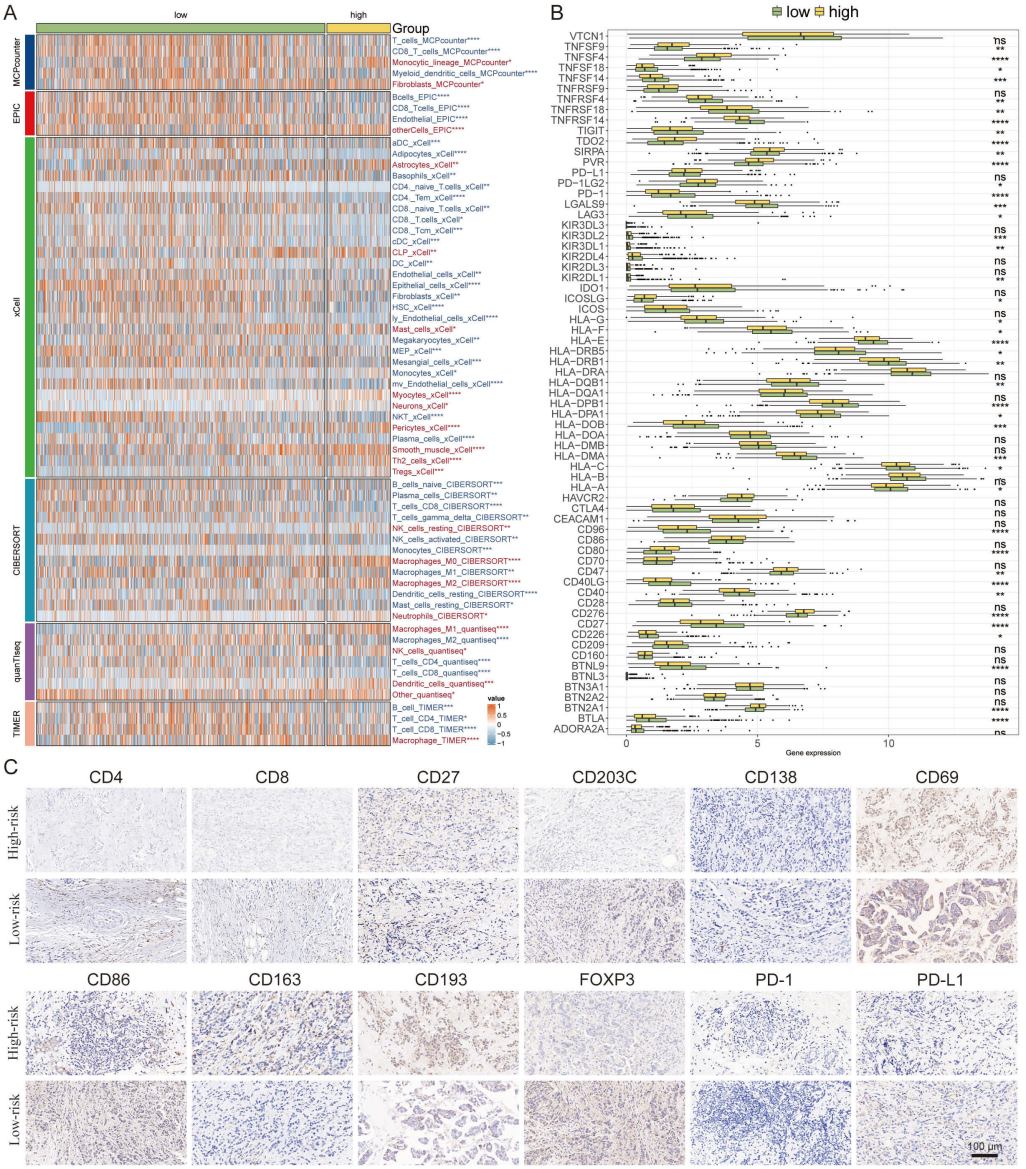
Delineation of the Immune Infiltration Landscape in Breast Cancer. **(A)** Heatmap generated using various computational algorithms (MCPcounter, EPIC, xCell, CIBERSORT, quanTIseq, TIMER) showing the immune cell infiltration landscape across low-risk and high-risk breast cancer groups. **(B)** Bar graph representation of cytokine profiles linked to immunoinfiltrating cells. **(C)** IHC staining panels of key immune markers. *P < 0.05, **P < 0.01, ***P < 0.001, ****P < 0.0001.

We embarked on a detailed exploration of the immune infiltrate landscape within breast cancer, employing a suite of computational algorithms—MCPcounter, EPIC, xCell, CIBERSORT, quanTIseq, and TIMER. This analysis illuminated distinct immune profiles between high and low-risk groups. The high-risk group was characterized by a pronounced infiltration of CD8 T cells and M2 macrophages, while the low-risk group displayed a notable presence of M1 macrophages and plasma cells (**Figure 7A**).

Furthermore, we charted the cytokine milieu associated with these immunoinfiltrates, discerning an increased expression of PD-1 within the low-risk group, which denotes a potential for a broader spectrum of therapeutic targets and possibly superior treatment outcomes (**Figure 7B**).

To validate and deepen our understanding of the TME, we conducted IHC staining of pivotal cellular markers and immune checkpoints. The representative staining of these IHC samples provided visual confirmation of the immune cell distributions (**Figures 7C**). The findings here reinforce the significance of immune cell profiles in determining patient risk stratification and underscore the potential for targeted immunotherapy approaches based on individual risk group categorizations.

### Forecasting immunotherapy outcomes with the VM-model

**Figure 8:**
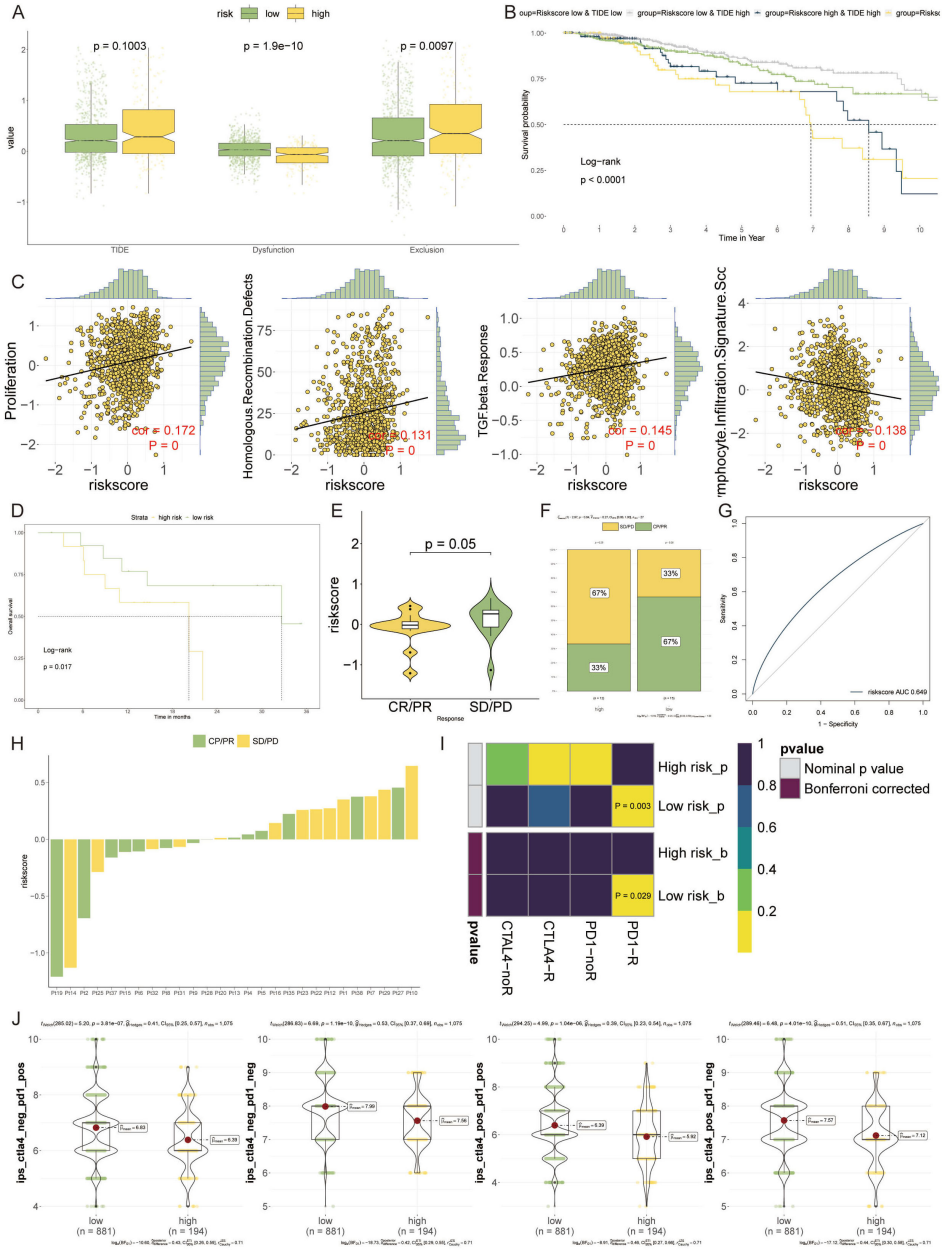
Forecasting Immunotherapy Outcomes with the VM-model. **(A)** Box plots depicting levels of TIDE, demonstrating increased immune evasion in the high-risk group compared to the low-risk group. **(B)** Kaplan-Meier survival curves illustrating the impact of TIDE and risk score on patient prognosis, with low-risk and lower TIDE scores associated with better outcomes. **(C)** Scatter plots showing the correlation of the risk score with various cellular processes. **(D)** Survival analysis of patients treated with PD-1 blockade therapy, highlighting improved outcomes in the low-risk group. **(E, F)** Violin plots and bar graphs assessing therapeutic benefits associated with PD-1 inhibitors, indicating a lower risk score is correlated with better therapeutic responses, especially in the low-risk group. **(G)** A ROC curve analysis presenting the relationship between PD-1 expression and VM-model risk score, suggesting the potential for identifying likely responders to PD-1 blockade therapy. **(H)** A horizontal bar chart visualizing the differential response to PD-1 immunotherapy across the patient cohort, emphasizing the diversity in treatment response. **(I)** Heatmap derived from SubMap algorithm analysis, revealing that low-risk patients are more responsive to PD-1 blockade treatments. **(J)** Bee-swarm plots showcasing the enhanced potential for response to anti-PD-1/PD-L1 and anti-CTLA-4 therapies in the low-risk group.

The potential of the VM-model to predict responses to immunotherapy was probed. Differential levels of TIDE suggested higher levels of immune evasion in the high-risk group, though this did not extend to the exclusion metric (**Figure 8A**). Prognostically, patients with higher TIDE scores yet lower risk scores showcased the most favorable outcomes (**Figure 8B**). Additionally, there was a significant positive correlation between the risk score and proliferation, homologous recombination defects, and TGF-beta response, while lymphocyte infiltration signatures were inversely related (**Figure 8C**).

The probability of survival following PD-1 blockade therapies was higher in the low-risk group, suggesting an inverse relationship with the VM-based risk score (**Figure 8D**). Exploring the therapeutic outcomes further, a lower risk score correlated with a therapeutic benefit, particularly in response to PD-1 inhibitors (**Figure 8E**). The high-risk group fared poorly in terms of treatment efficacy, whereas the low-risk group showed a clear treatment advantage (**Figure 8F**). The risk score showed a positive correlation with PD-1 expression, suggesting its utility in identifying potential beneficiaries of PD-1 blockade therapy (**Figure 8G**). A distribution map pinpointed the differences in PD-1 immunotherapy responses between individual patients, underlining the variability within the cohort (**Figure 8H**). Over the past years, immune checkpoint inhibitors targeting PD-1, such as pembrolizumab and nivolumab, and CTLA-4, like Tremzumab, have been integrated into the immunotherapeutic landscape. However, their efficacy in solid tumors, breast cancer included, has been modest.

To evaluate the prognostic value for immune checkpoint blockade response, we employed SubMap algorithms stratified by VM-model. Notably, patients with low-risk appeared more responsive to PD-1 blockade (Bonferroni corrected p = 0.029) (**Figure 8I**). Furthermore, the low-risk group manifested a markedly enhanced potential for response to both anti-PD-1/PD-L1 and anti-CTLA-4 treatments (**Figure 8J**).

### Strategic selection of therapeutic targets and agents for high-risk VM patients

**Figure 9:**
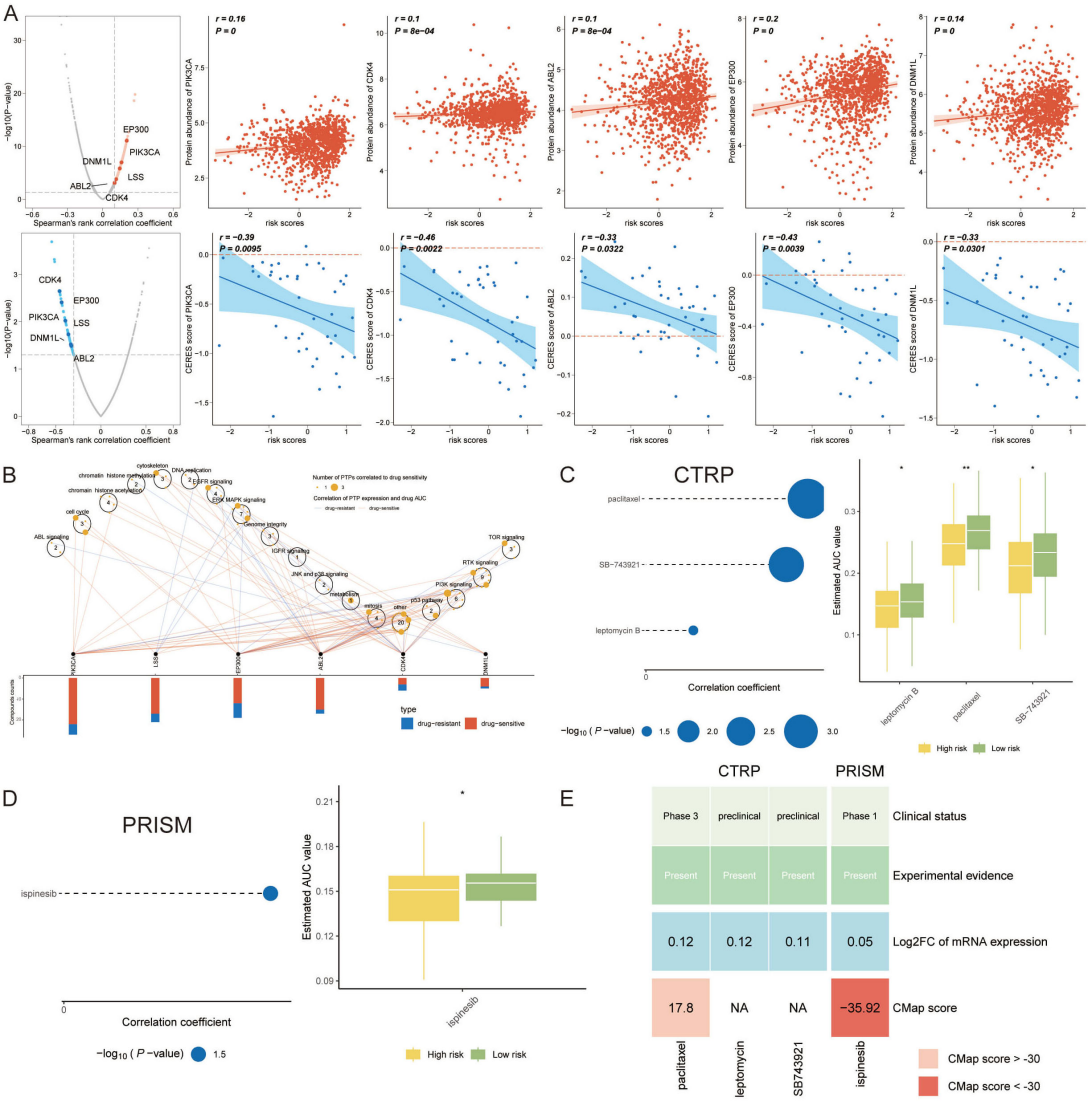
Strategic Selection of Therapeutic Targets and Agents for High-Risk VM Patients. **(A)** Scatter plots detailing the correlation between VM risk scores and the expression levels of potential druggable genes, with a focus on those exceeding a correlation coefficient threshold of 0.20 (p < 0.05), indicating a significant relationship with prognostic risk. **(B)** Network graph of the identified targets, overlaid with drug sensitivity data, showing which genes are most responsive to existing therapeutic compounds, thereby spotlighting five key genes as high-value targets for therapeutic intervention. **(C)** Bubble chart from the CTRP demonstrating the correlation between gene targets and drug sensitivity, with larger bubbles representing a stronger relationship indicative of potential drug efficacy. *P < 0.05, **P < 0.01. **(D)** Box plots comparing the estimated area under the dose-response curve (AUC) for four selected compounds between high-risk and low-risk groups, revealing lower AUC values for the high-risk group, suggesting these patients may derive more benefit from these drugs. *P < 0.05. **(E)** Bar chart juxtaposing clinical trial status and experimental evidence from literature with CMap scores, where ispinesib shows a highly negative CMap score, suggesting a significant potential for reversing breast cancer-specific gene expression patterns.

In our quest to discover actionable therapeutic targets for high-risk breast cancer patients with poor prognoses, we embarked on a methodical compilation of target data for 6,125 compounds. A two-phased analytical process ensued to discern viable candidates. Initially, we calculated the correlation coefficients to gauge the interplay between druggable gene expression levels and VM-based risk scores, isolating 246 gene targets with coefficients exceeding 0.20 (p < 0.05). Subsequently, correlation analyses on high-risk breast cancer cell lines honed in on 74 targets with poor prognostic dependencies, as evidenced by their CERES scores aligned with risk scores. As a result, a total of six gene targets were generated that met the two conditions of the appeal (**Figure 9A**).

This approach spotlighted five genes as prime therapeutic targets, suggesting that disruptions in these genes might offer clinical benefits to breast cancer individuals. Evaluations of drug sensitivity further distilled the list to five genes, markedly responsive to therapeutic agents (**Figure 9B**). Drug response analyses indicated four compounds—paclitaxel, SB-743921, leptomycin B, and ispinesib—garnered lower estimated AUC values in high-risk cohorts, hinting at a greater ameliorative potential for these patients (**Figures 9C, D**).

We undertook a comprehensive analysis encompassing clinical status and experimental evidence, referencing PubMed, to ascertain the most viable therapeutic options among the candidates. Enhanced fold changes in gene expression levels hinted at these compounds’ therapeutic potency for breast cancer treatment. Complementary to this, Connectivity Map (CMap) analysis was employed to filter for compounds exhibiting gene expression profiles antithetical to breast cancer-specific patterns. Notably, ispinesib manifested a CMap score surpassing -35, delineating it as a particularly potent candidate (**Figure 9E**). This integrative approach ensures that the highlighted therapeutic agents not only demonstrate strong *in vitro* efficacy but also possess substantial clinical and preclinical backing.

## Discussion

Breast cancer has a high incidence and mortality rate worldwide, especially among women, is the most common cancer, and current treatments for breast cancer include targeted therapy, hormone therapy, and radiation therapy (35, 36). Surgery is an important treatment strategy for individuals whose breast cancer has not yet spread to other parts of the body (37). But in most women, the absence of breasts can lead to feelings of asexuality and loss of self-image, which can lead to depression (38). So clinicians and surgeons focus not only on the tumor-specific characteristics of breast cancer, but also on patient function, tolerance, comorbidities, and life expectancy to determine the best treatment (39). Therefore, it is necessary to find new factors to predict the prognosis of breast cancer patients. The integration of ML techniques to unravel the complexity of VM in breast cancer marks a pivotal advancement in our understanding of tumor biology. Our findings underscore the significant potential of ML-driven models to predict breast cancer outcomes by identifying a VM signature. This approach not only enhances our grasp of VM’s role in cancer progression but also opens new avenues for targeted therapy development.

Our study focused on identifying VM-associated genes, which are critical for the formation of vascular mimicry structures within tumors. Using machine learning algorithms on multi-omics data from over 6,000 breast cancer patients, we identified six key genes significantly associated with VM and patient risk scores: EP300, PIK3CA, DMXL1, LS, ABL2, and CDK4. These genes serve as potential biomarkers for high-risk patients, indicating a more aggressive cancer phenotype.

In the context of previous research, our findings align with studies highlighting the role of PIK3CA and EP300 in tumor progression and VM formation. PIK3CA mutations are known to activate the PI3K/AKT signaling pathway, promoting cell proliferation and survival, which are crucial for VM structures (40, 41). EP300, a histone acetyltransferase, regulates gene expression involved in cell cycle and differentiation, further supporting its association with VM (42).

Our analysis further revealed significant differences in the tumor microenvironment between high-risk and low-risk groups. High-risk patients exhibited altered interactions, particularly involving macrophages and plasma cells, which are known to influence tumor progression and immune evasion. These findings emphasize the role of immune cells in the tumor microenvironment, with macrophages (especially tumor-associated macrophages or TAMs) contributing to an immunosuppressive environment that promotes tumor growth and metastasis (43, 44). High-risk patients displayed a higher TMB compared to low-risk patients. This is significant as increased TMB is often associated with better responses to immunotherapy due to higher neoantigen load and potential immune recognition (45). The differential expression patterns of VM-related genes in high-risk groups underscore the aggressive nature of these tumors and highlight the importance of targeting these molecular alterations for effective treatment.

In the second phase of our study, we evaluated the sensitivity of VM-related genes to various compounds. This analysis identified four drugs—paclitaxel, SB-743921, leptomycin B, and ispinesib—as promising candidates due to their significant efficacy in targeting high-risk breast cancer cells. Notably, ispinesib emerged as the most promising candidate due to its high specificity and therapeutic potency. Ispinesib’s mode of action as a kinesin spindle protein (KSP) inhibitor effectively targets rapidly proliferating tumor cells involved in VM. This mechanistic insight aligns with previous studies demonstrating the efficacy of KSP inhibitors in reducing tumor growth and enhancing apoptosis (46). The CMap analysis further validated ispinesib’s potential, revealing a gene expression profile antithetical to breast cancer-specific patterns. To validate our findings, we conducted an extensive literature review and performed CMap analysis. The CMap scores for ispinesib and other compounds confirmed their potential as effective treatments for high-risk VM patients. The integration of clinical trial data from PubMed further reinforced the therapeutic relevance of these drugs, highlighting their potential to improve patient outcomes. Paclitaxel is widely used in clinical settings, while ispinesib and SB-743921 have shown promise in preclinical and early clinical trials, underscoring their potential for clinical application (47).

While our model demonstrates robust predictive power, it’s essential to acknowledge the limitations inherent in our study. First, the reliance on single-cell RNA sequencing data, while providing unparalleled resolution of tumor heterogeneity, may not capture the entire spectrum of VM characteristics. Factors such as tumor microenvironmental variations and the dynamic nature of VM over the disease course pose challenges to model generalization. Additionally, the performance of ML models, including ours, hinges on the quality and diversity of the training data. Thus, our model’s applicability to broader patient populations requires validation across diverse datasets.

Another critical consideration is the assumption that VM’s genomic signature remains constant across different stages of breast cancer. This assumption, while necessary for model development, may oversimplify the dynamic interplay between tumor cells and their environment. Future iterations of our model should incorporate temporal data to capture the evolution of VM signatures over time.

In our previous work (6), we developed a predictive model for breast cancer prognosis based on endoplasmic reticulum (ER) stress-related genes. This study highlighted the critical role of ER stress in tumor progression and response to therapy. The current study advances this field by focusing on VM, a distinct mechanism by which tumor cells mimic endothelial cells to form vasculogenic-like networks, contributing to tumor growth and metastasis. The novel VM model demonstrates robust predictive power, with a significant improvement over traditional prognostic method. The model’s ability to stratify patients based on VM activity provides a nuanced understanding of tumor biology, leading to more personalized treatment approaches.

## Conclusion

Our study represents a significant leap forward in the application of ML to cancer research, specifically in the context of VM in breast cancer. While acknowledging the limitations and assumptions of our current model, we emphasize the vast potential of this research to impact future diagnostic and therapeutic strategies. The road ahead calls for collaborative efforts across computational and clinical disciplines to refine, validate, and translate these findings into clinical practice, ultimately aiming to improve outcomes for breast cancer patients worldwide.

## Data availability statement

The datasets presented in this study can be found in online repositories. The names of the repository/repositories and accession number(s) can be found in the article/**Supplementary Material**.

## Ethics statement

The studies involving humans were approved by the Ethics Committee of Guizhou Provincial People’s Hospital (2023-070). The studies were conducted in accordance with the local legislation and institutional requirements. The participants provided their written informed consent to participate in this study.

## Author contributions

XL(1^st^ author): Data curation, Formal analysis, Investigation, Visualization, Writing – original draft. XL(2^nd^ author): Investigation, Resources, Visualization, Writing – original draft. BY: Methodology, Resources, Writing – original draft. SS: Investigation, Resources, Writing – original draft. SW: Investigation, Resources, Writing – original draft. FY: Conceptualization, Methodology, Supervision, Writing – review & editing. TW: Conceptualization, Formal analysis, Investigation, Methodology, Resources, Validation, Visualization, Writing – original draft, Writing – review & editing.
